# *De novo* reconstruction of the *Toxoplasma gondii* transcriptome improves on the current genome annotation and reveals alternatively spliced transcripts and putative long non-coding RNAs

**DOI:** 10.1186/1471-2164-13-696

**Published:** 2012-12-12

**Authors:** Musa A Hassan, Mariane B Melo, Brian Haas, Kirk D C Jensen, Jeroen P J Saeij

**Affiliations:** 1 Department of Biology, Massachusetts Institute of Technology, Cambridge, Massachusetts, USA; 2 Genome Annotation Research and Development, Broad Institute of Harvard and MIT, Cambridge, Massachusetts, USA

**Keywords:** *Toxoplasma*, RNA-seq, Trinity, LincRNA, Alternative splicing, Transcriptome

## Abstract

**Background:**

Accurate gene model predictions and annotation of alternative splicing events are imperative for genomic studies in organisms that contain genes with multiple exons. Currently most gene models for the intracellular parasite, *Toxoplasma gondii*, are based on computer model predictions without cDNA sequence verification. Additionally, the nature and extent of alternative splicing in *Toxoplasma gondii* is unknown. In this study, we used *de novo* transcript assembly and the published type II (ME49) genomic sequence to quantify the extent of alternative splicing in *Toxoplasma* and to improve the current *Toxoplasma* gene annotations.

**Results:**

We used high-throughput RNA-sequencing data to assemble full-length transcripts, independently of a reference genome, followed by gene annotation based on the ME49 genome. We assembled 13,533 transcripts overlapping with known ME49 genes in ToxoDB and then used this set to; a) improve the annotation in the untranslated regions of ToxoDB genes, b) identify novel exons within protein-coding ToxoDB genes, and c) report on 50 previously unidentified alternatively spliced transcripts. Additionally, we assembled a set of 2,930 transcripts not overlapping with any known ME49 genes in ToxoDB. From this set, we have identified 118 new ME49 genes, 18 novel *Toxoplasma* genes, and putative non-coding RNAs.

**Conclusion:**

RNA-seq data and *de novo* transcript assembly provide a robust way to update incompletely annotated genomes, like the *Toxoplasma* genome. We have used RNA-seq to improve the annotation of several *Toxoplasma* genes, identify alternatively spliced genes, novel genes, novel exons, and putative non-coding RNAs.

## Background

*Toxoplasma gondii* is a highly prevalent obligate intracellular protozoan parasite causing disease in immunocompromised individuals and congenitally infected infants. Ten *Toxoplasma* strains, representing predominant strains in Europe, North and South America [1,2] and a type II/III recombinant strain [3] have been sequenced, with the ME49, a type II strain, genome used as a reference. The *Toxoplasma* genome, which is publicly available in the *Toxoplasma* database (ToxoDB), is approximately 65Mb, made up of 14 chromosomes, and 8155 genes, with an average of 4.1 introns per gene and a 52% G / C content [4].

While computational tools such as GlimmerHHM and TigrScan [5], and TwinScan [6] have been useful resources for *Toxoplasma* gene model predictions, differences in the algorithms used by these programs have often resulted in different gene models, leading to uncertainties in the current gene models [7]. Accurately annotated gene models are imperative for genomic research on *Toxoplasma* but sufficient genomic data, such as full-length complementary DNA (cDNA) sequences, is not available to refine the computationally predicted gene models. Additionally, even though there are reports of alternative splicing of some *Toxoplasma* genes [8-10], it is currently unknown what the extent of alternative splicing is in *Toxoplasma*. Transcript sequence data, such as expressed sequence tags (ESTs), full-length cDNAs (FL-cDNAs), and cDNA sequences, provide reliable evidence for resolving gene structures [11] because they define the intron-exon boundaries and are similar to the genomic sequence. FL-cDNAs would be ideal for gene annotation, since they encode the full-length transcript with well demarcated exon-exon junctions, but the current cost of Sanger sequencing makes this method very expensive. Although there is an abundance of EST and end-sequenced cDNA data (mostly used for UTR annotations) [4,7,12,13], few FL-cDNA sequence data is available for *Toxoplasma*. RNA sequencing (RNA-seq) [12,14-16] which generates short cDNA sequences (shorter than ESTs), has become a powerful tool for gene expression studies and for the *de novo* assembly of transcriptomes [17-19]. The short sequences generated by RNA-seq, however, must first be assembled into full transcript structures using either of two strategies [20,21]: 1) ‘mapping-first’ strategy [22-24], which involves first aligning the short reads to a reference genome followed by merging of sequences with overlapping alignments, and spanning splice junctions, or 2) ‘assembly-first’ (*de novo*) strategy [17-19], which uses the reads to directly assemble transcripts that can then be mapped to a reference genome.

Generally any approach used to reconstruct transcripts from RNA-seq data must be able to navigate the complications imposed by: i) low expression of some genes that are in turn represented by low RNA-seq data thus making them difficult to fully reconstruct, and ii) short read lengths and alternative splicing which often makes it hard to correctly match each isoform with a read [21]. While the ‘mapping first’ approach is ideal for model organisms with complete genomes, it still requires correct alignment of the reads to the reference genome, a fact that is convoluted by alternative splicing and sequencing errors. Furthermore, even in well annotated genomes, like the mouse and human genomes, there are still novel gene annotations being discovered [23], making it imprudent to completely rely on currently available gene structures. Nevertheless, this approach promises maximal sensitivity and requires less computational resources compared to the assembly first approach. On the other hand since the ‘assembly first’ approach does not require a reference genome, it is well suited for reconstructing transcripts from organisms with incomplete or no annotated genomes. However, besides the large amount of computational infrastructure required, the key challenge to this approach is the partitioning of reads into components representing transcript isoforms. That is, as the number of reads increases, it becomes difficult to determine which reads should be joined into a contiguous contig. However, this problem has been resolved by the use of the *de Bruijn* graph which models overlapping sequences rather than reads, thereby reducing the complexity of dealing with multiple reads [25-27]. Additionally, by analyzing the graph paths taken by the reads and read pairs and applying a coverage cutoff to determine which path to follow or to remove [18,21], the problem posed by sequencing errors from variations, which can make the graph complex by introducing branching points are easily resolved [21]. Overall, both of these approaches have been reported to accurately reconstruct several transcripts and alternative isoforms [23,24,28], therefore, the choice of which method to use is invariably dependent on the availability of a well annotated reference genome and the biological question to be answered.

We sequenced cDNA from polyadenylated RNA obtained from murine bone marrow derived macrophages infected with a type II *Toxoplasma* strain (Pru). Because currently there is no annotated genome for the Pru strain, and the ME49 reference genome is not complete, we then used Trinity-based *de novo* transcript assembly [18] to reconstruct transcripts from approximately 270 million RNA-seq reads. Finally, we used PASA (Program to Assemble Spliced Alignments) [29-31], and the published ME49 reference genome (ToxoDB) [4], to filter invalid assemblies and transcripts likely resulting from sequencing errors, and to reconstruct more complete transcripts. PASA assembles overlapping and compatible alignments, which are defined as overlapping alignments transcribed on the same strand and have identical introns in the regions of their overlap [31], and is therefore suitable for the discovery of alternative splicing variants and novel transcripts. Transcripts derived from *de novo* assembly and alignments to a reference genome are reported to provide a template for genome annotation that compares well with the utility of FL-cDNAs [11]. Using this hybrid approach (*de novo* assembly followed by alignment to a reference genome), we provide transcript-based gene structures that we then use to confirm ToxoDB gene models, update existing UTRs, update current ToxoDB predicted intron/exon boundaries, identify new gene models that represent alternatively spliced isoforms, and identify new genes. In summary, we have identified 2,930 transcripts not overlapping with any known ME49 genes; some of which may be novel genes, or non-coding RNAs. Additionally, we have identified 50 alternatively spliced transcripts and report on their differential usage amongst 3 clonal *Toxoplasma* strains.

## Results and discussion

### *De novo* full-length transcript assembly

**Figure 1:**
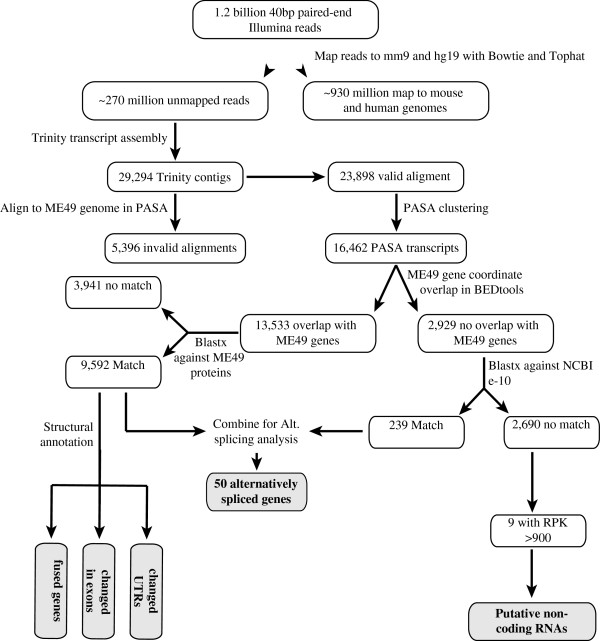
**Schematic of methods used for *de novo *transcript assembly and genome annotation with RNA-seq.** A workflow of the strategy used to assemble and annotate *Toxoplasma* full-length transcripts using RNA-seq data. Because genes with updated annotations may fall into multiple categories (i.e. fused genes, changed exons, and changed UTRs) we highlight only the types of variation observed in the current study.

Approximately 1.2 billion 40 base-pair (bp) paired-end RNA-seq reads generated from murine bone-marrow derived macrophages infected with *Toxoplasma* were used to assemble *Toxoplasma* full-length transcripts in Trinity [18] and PASA [29,31]. A flow chart of the steps followed to assemble and annotate transcripts is presented in Figure 1. Because the parasites used to infect the murine macrophages were grown in human foreskin fibroblasts (HFFs), we initially used the genome alignment tool, Tophat [32,33] to sequentially align the RNA-seq reads to the mouse (mm9) and human (hg19) reference genomes and a collection of mouse and human splice junctions [34], to filter out mouse and human reads. Since the two genomes (mouse and human) are almost complete, about 270 million reads, which did not align to them, were considered to be mainly *Toxoplasma* derived and were used as input for *de novo* transcript assembly in Trinity (Materials and Methods). In total, we assembled 29,294 contigs (hereafter referred to as Trinity contigs).

The transcript assembly strategy employed in Trinity (detailed in Grabherr *et al*. [18]) requires Trinity contigs to be joined only if there is a substantial overlap between them. Therefore, reads obtained from an alternatively spliced gene will be assembled into one complete transcript and one contig representing the unique region from the second isoform. Additionally, lowly expressed genes may not generate enough RNA-seq reads for complete transcript assembly, particularly for longer genes. Consequently, the Trinity contigs will be a mixture of complete transcripts (includes one isoform of each alternatively spliced gene), unique regions of alternatively spliced transcripts, and fragments from lowly expressed genes. Thus, to filter Trinity contigs and identify alternatively spliced transcripts, we used PASA to assemble and cluster them based on the positions they aligned to in the type II (ME49) *Toxoplasma* genome. Following assembly in PASA, 23,898 out of the 29,294 Trinity contigs (81%) had valid ME49 genome alignments and were clustered into 16,462 transcripts (henceforth PASA transcripts), while 5,396 had invalid alignments and were excluded from PASA analysis (Additional files 1, 2, 3 and 4).

A major limitation to both *de novo* and *ab initio* transcriptome assembly is that overlapping transcripts can be joined even though they are not from polycistronic RNA (reviewed in [35]). For overlapping transcripts transcribed on opposite strands, this problem can be mitigated by the use of strand specific RNA-sequencing [36,37]. However, overlapping transcripts transcribed from the same strand can only be separated by using cap- or end-specific RNA-seq [35]. However, amongst the requirements for valid transcript alignment to the genome in PASA is that all inferred exon-intron boundaries must have consensus splice sites, a requirement that is unlikely to be met when overlapping UTRs from two adjacent genes translated on opposite strands are joined in Trinity to form a spurious intron. Furthermore, during transcript annotation update in PASA, some fused transcripts will be rejected if considered to result in transcripts that are out of frame with the existing gene structures. Additionally, transcripts can only have valid alignments in PASA if 90% of their length has 95% sequence similarity with the ME49 genome. Consequently, we postulated that some of the 5,396 Trinity contigs that did not have valid alignments to the ME49 genome in PASA contain *Toxoplasma* transcripts that were erroneously joined in Trinity due to overlapping UTRs and transcripts that did not meet the 90% length requirement. Indeed when these 5,396 Trinity contigs were aligned to ME49 proteins using Blastx [38], 749 matched known ME49 proteins. We envisioned two possible scenarios for overlapping genes joined by chance in Trintiy: a) if two fully assembled transcripts are joined due to overlapping UTRs the resulting transcript will produce 2 ORFs, each significantly matching either one of the two overlapping genes, and b) if only one transcript of the joined pair is completely assembled then it will align to the protein sequence of only one of the genes. Of the 749 Trinity contigs having significant matches against ME49 genes in Blastx, 95 significantly matched at least 2 adjacent ME49 genes (Additional file 5). An example is the Trinity contig comp489_c0_seq1, which significantly matched three adjacent genes; *Rop18* (TGME49_005250), TGME49_005240, and TGME49_005230, but produced only 2 ORFs one matching *Rop18* and the other matching TGME49_005240 and TGME49_005230. Thus, TGME49_005240 and TGME49_005230 seem to be a single gene but the 3’UTR of TGME49_005240 gene was erroneously joined with the 5’ UTR of *Rop18*. Indeed splice junction tracks, supported by RNA-seq data, available in ToxoDB confirm the existence of a splice site between the last exon of TGME49_005230 and the first exon of TGME49_005240 (Additional file 6). We conclude that these 95 transcripts were erroneously joined in Trinity due to an overlap in their UTRs and were therefore, rejected by PASA. The rest of the 654 Trinity contigs that aligned to ME49 proteins are *Toxoplasma* transcripts that were discarded in PASA because they did not meet the 90% alignment length requirement imposed in PASA. The other 4,647 Trinity contigs that did not match ME49 genes produced significant matches against mouse and human bacterial artificial clone (BAC) sequences available in the NCBI non-redundant nucleotide database, and none mapped to known mouse or human genes.

We hypothesized that the limiting factor for full-length transcript assembly is the RNA-seq coverage of each gene. To test this, we calculated the number of RNA-seq reads overlapping each full-length PASA transcript (we define full-length PASA transcripts in this case as transcripts translated into proteins with identical sequence and length to those predicted in ToxoDB) then binned the PASA transcripts based on RNA-read coverage. Because there is a high probability of sequencing cDNAs from long compared to short transcripts, reads from longer transcripts tend to be over-represented in the sequencing data. We correct for this bias by presenting coverage as a fraction of transcript length, reads per kilobase (RPK). We have also provided the raw read coverage for each PASA transcript (Additional file 7). As expected, we found that the ability to assemble complete transcripts was improved with high read coverage of each gene (Additional file 8).

### Identification of novel *Toxoplasma* genes

**Figure 2:**
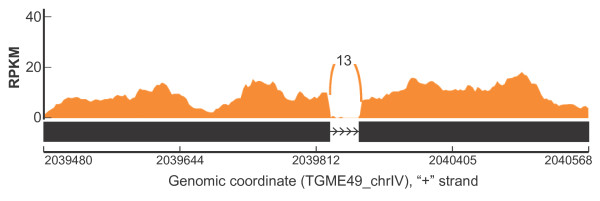
**2,930 PASA transcripts did not overlap with any of the currently annotated (ToxoDB) type II (ME49) *Toxoplasma *genes.** Shown is a PASA transcript S2826 (Black) that aligned to a genomic sequence on TGME49_chrIV but did not overlap with any of the ME49 genes. We also show RNA-seq reads pile-up (histogram represented as reads per kilobase per million reads or RPKM) on the exons and 13 reads mapping to the exon-exon junction. This transcript produces an ORF of 146 amino acids, which is homologous to the TGGT1_124090 protein from the type 1 GT1 strain, but has no homology to any annotated ME49 protein.

Since the *Toxoplasma* genome annotation is incomplete, we postulated that some of the PASA transcripts were putative novel ME49 and *Toxoplasma* genes (we define novel ME49 genes as genes already described in other *Toxoplasma* strains but not in ME49 and novel *Toxoplasma* genes as those yet to be annotated in any *Toxoplasma* strain in ToxoDB). Because the PASA transcripts are annotated based on the region they aligned to in the ME49 genome, we searched for PASA transcripts whose genomic coordinates overlapped with known ME49 genes in ToxoDB (hereafter ToxoDB genes) using BEDtools [39]. We confirmed some of these intersections by simultaneous viewing of individual PASA transcripts and ToxoDB genes in the Integrative Genomics Viewer (IGV) [40]. We identified a total 13,533 PASA transcripts with, and 2,929 PASA transcripts without genomic coordinate overlap with known ToxoDB genes (Additional file 7). Approximately, 93% of the 2,929 transcripts were predicted to be from single exon genes, and 65 (2%) aligned to sequences on the ToxoDB DS984XXX-designated scaffolds, corresponding to scaffolds yet to be assigned to the 14 *Toxoplasma* chromosomes. Because the 2,929 transcripts align to the ME49 genome but do not overlap any known ME49 gene, and have substantial raw RNA-seq reads pile-up (Figure 2), they are potentially transcribed from novel genes.

To investigate if any of these PASA transcripts were indeed novel protein coding transcripts, we performed a Blastx search against the non-redundant protein database in NCBI ( http://www.ncbi.nih.gov), with an expectation cutoff of 10^-10^. 27 of these transcripts matched known ME49 proteins and were excluded from potential novel genes. We postulate that these 27 transcripts may be novel genes but produce ORFs that are partially identical to other known ME49 proteins. 212 of the remaining 2902 transcripts had significant matches against other proteins in the non-redundant NCBI database. Interestingly, the majority of the transcripts, 202, significantly matched sequences from either *Neospora caninum,* a close relative of *Toxoplasma,* or from other *Toxoplasma* strains. 18 transcripts out of the 212 were of the same length and had identical sequences to proteins already described in other *Toxoplasma* strains (VEG and GT1) indicating that these are genes yet to be annotated in the ME49 strain (novel ME49 genes); the strain to which we aligned the PASA transcripts (Additional file 9). While another 16 transcripts significantly matched *Toxoplasma* proteins of the same length in the NCBI database, the percentage of sequence similarity ranged from 91-99%. Potentially, these 16 are novel ME49 genes, but unlike the first 18, are polymorphic between ME49 and the other *Toxoplasma* strains. An example of this is a 125 amino acid long protein, which produced two significant hits against GT1 (TGGT1_064380) and VEG (TGVEG_059280) proteins with three mismatches; two mismatches occurring at positions where the GT1 and VEG proteins showed polymorphism, and the other mismatch was at a position where the GT1 and VEG proteins were homologous. The other proteins that aligned to *Neospora* proteins may be proteins yet to be described in any of the *Toxoplasma* strain i.e. novel *Toxoplasma* genes.

The remaining 2,690 PASA transcripts that produced no significant Blastx match are unlikely to encode proteins and are potential non-coding RNAs or spurious transcript assemblies. However, some of these transcripts could be fragments from novel genes and therefore not all of them can be regarded as non-coding RNAs. Nevertheless, about 80% of PASA transcripts with RPK values above 900 were fully assembled (Additional file 8). We therefore, investigated the likelihood of the 2,690 PASA transcripts being pieces of incompletely assembled novel genes based on their RPK values. Of the 2,690 PASA transcripts, 9 (6 of which have a single exon) had RPK values above 900 and a minimum length of 200bp (in mice and humans lincRNAs are defined as non-coding transcripts longer than 200bp [41,42]). Even though majority of the 9 transcripts have single exons, based on the RPK values, these 9 transcripts are likely to be fully assembled. Because lincRNAs are often expressed at low levels compared to protein-coding genes [24,41,43], deeper sequencing might clarify if the other transcripts are fragments of new genes or are putative lincRNAs.

### *Toxoplasma gondii* gene model annotation

Following the identification of transcripts overlapping known ME49 genes, we compared our predicted PASA gene models and the current ToxoDB gene models. Even though each PASA transcript overlapping with a ME49 gene is likely transcribed from that gene, some of these PASA transcripts could be fragments sequenced from retained introns, misassemblies, antisense transcripts, misalignments, or non-coding RNAs transcribed from within known genes. Consequently, to identify which of the 13,533 PASA transcripts overlapping with known ME49 genes were protein-coding, we performed a Blastx search against known ME49 proteins, with an expectation cutoff of 10^-10^; 9,592 non-redundant PASA transcripts produced significant matches, with 2,073 transcripts producing ORFs of identical length and sequence to the protein from the ME49 gene they overlapped. The remaining 3,941 did not produce any significant match and were excluded from further analysis. It is plausible that the PASA transcripts with no significant match are fragments from larger genes not fully reconstructed due to insufficient RNA-seq read coverage. However, about 9 of these transcripts had RPK values >900, the level at which about 80% of the transcripts were fully assembled (Additional file 8). Consequently, we propose that some of these transcripts are putative non-coding RNAs transcribed from within known *Toxoplasma* genes, are antisense RNA (aRNA), are translated on different frames from those used in predicting ToxoDB proteins, or that some of the currently predicted ToxoDB genes overlapped by these PASA transcripts are spurious.

**Figure 3:**
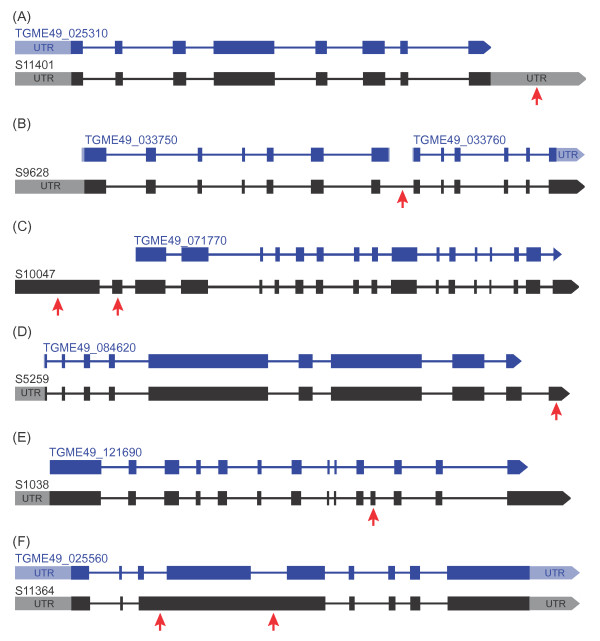
**Refinement of gene models as currently predicted in ToxoDB.** (**A**) A ToxoDB gene (Blue) with discrepancies in the UTRs compared to the PASA transcript (Black). (**B**) Two ME49 genes (Blue) are fused into one PASA transcript (Black). (**C**) A PASA transcript with two novel 5’ exons lacking in the predicted ME49 gene (Black). (**D**) A PASA transcript (Blue) with a novel 3’ exon lacking in the ME49 gene (Blue). (**E**) A PASA transcript (Blue) with a novel internal exon in a region containing an intron in a ME49 gene (Black). (**F**) A ME49 gene (Black) with three exons fused into one in the corresponding PASA transcript (Blue)

When we compared PASA and ToxoBD gene models, we found inconsistencies which we classified as follows:

a) Variations in ToxoDB UTRs annotation: Inaccuracies in the UTRs of annotated ToxoDB genes has previously been reported [7]. This set constituted the majority of variation evidenced between our predicted models and those available in ToxoDB. Since computational models, which is the basis for most of the ToxoDB gene models, are based on the prediction of coding sequences (CDS) while transcript-based models rely on the whole RNA, variations in UTRs between computational and RNA-seq predicted gene models are expected to dominate. This set will produce ORFs with identical sequence and length to the proteins of ToxoDB transcripts they overlapped, but will have different gene coordinates due to differences in UTR lengths (Figure 3a).

b) Fused genes: This category includes transcripts predicted in PASA to be single genes but are reported in ToxoDB to be more than one individual gene. As indicated above, genes transcribed from the same or opposite strands that were spuriously joined due to overlapping UTRs did not pass the PASA filter. Consequently, the transcripts included in this set are those that had splice sites at the inferred exon-intron junctions, had RNA-seq reads supporting the splice junctions, and were in frame with the annotated ToxoDB genes that were predicted to be fused. About 74 transcripts were included in this category (Figure 3b).

c) ToxoDB genes predicted in PASA to have either different transcription start sites or novel 5’ exons: This group includes transcripts which even though they have ORFs that are identical in sequence to the protein of the ToxoDB gene they overlap, the ORFs differ in length from the ToxoDB protein. Amongst these were transcripts with extra 5’ exons not predicted in the ToxoDB gene they overlapped, and transcripts with the same exon counts to the ToxoDB gene but different 5’ start sites (Figure 3c).

d) Inaccuracies on the 3’ end sites of ToxoDB genes. These included ToxoDB genes predicted in PASA to have extra 3’ exons or different 3’ end sites (Figure 3d). Like c above, these transcripts produced ORFs that were identical to the proteins of the ToxoDB genes they overlapped but were of variable length either due to a novel 3’ exon or different 3’ end site.

e) Novel exons within predicted ToxoDB genes: We found transcripts with at least one novel exon in regions annotated for introns within protein-coding ToxoDB genes (Figure 3e).

f) Of the changed ToxoDB genes, we found genes with fewer or spurious exons compared to the PASA transcripts (Figure 3f).

It is worth noting that there are overlaps in the identities of ToxoDB genes grouped into these categories and that unlike inaccuracies in the UTRs, variations in the 5’ start and 3’ end sites result in proteins of different lengths to those predicted in PASA. Additionally, due to the possibility of some of the PASA transcripts being incomplete assemblies, we did not investigate longer ToxoDB genes that are potentially several genes fused together (the reverse of “b” above).

### Assessing alternative splicing in *Toxoplasma gondii*

**Figure 4:**
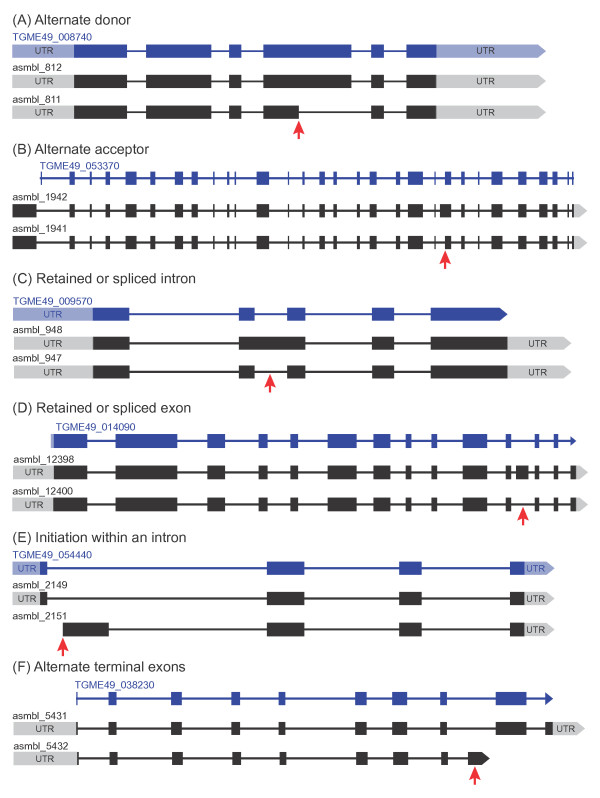
**Alternative splicing in *Toxoplasma *takes similar forms described in other eukaryotes.** Types of alternative splicing included Alternate Donor (**A**), Alternate Acceptor (**B**), Retained or Spliced Intron (**C**), Retained or Spliced Exons (**D**), Initiation Within and Intron (**E**) and Alternate Terminal Exons (**F**). ToxoDB genes are depicted in blue and PASA transcripts in black. Red arrows indicate regions where variation is observed.

To determine the extent of alternative splicing in *Toxoplasma*, we used the protein-encoding PASA transcripts overlapping (9,592) and not overlapping (239) with known ME49 genes. Transcripts were considered to be splice variants if they aligned to the same genomic locus, and were transcribed from the same stand. Because variants supported by a single RNA-seq read can be due to sequencing errors, reads from pre-processed RNA, and errors in transcription, we computed the number of reads supporting the regions unique to each isoform i.e. reads supporting the existence of an alternative isoform; all the remaining variants were supported by at least two unique reads. In total 77 genes were alternatively spliced, resulting in 152 transcripts. However, only 50 alternatively spliced genes resulted in different transcript and protein isoforms (Additional file 10). We did not observe any unique splicing patterns in the current study with splicing in *Toxoplasma* generally taking the forms previously described in other eukaryotes [44] and included 1) alternate acceptor (AA), 2) alternate donor (AD), 3) alternate terminal exon (ATE), 4) retained intron (RI), 5) spliced intron (SI), 6) skipped exon (SE), 7) retained exon (RE), and 8) initiation within intron (IWI), where RE and SE, and RI and SI are reciprocals. The description of each class is contained in Campbell *et al.*[31] and an example of each is presented in Figure 4. In our data, the majority of the alternative events (40%) belonged to the AD class. Additionally, because the isoform annotation is based on pooling of reads from 27 samples, the distribution of this splice isoforms amongst the individual samples is not apparent in the current study.

**Figure 5:**
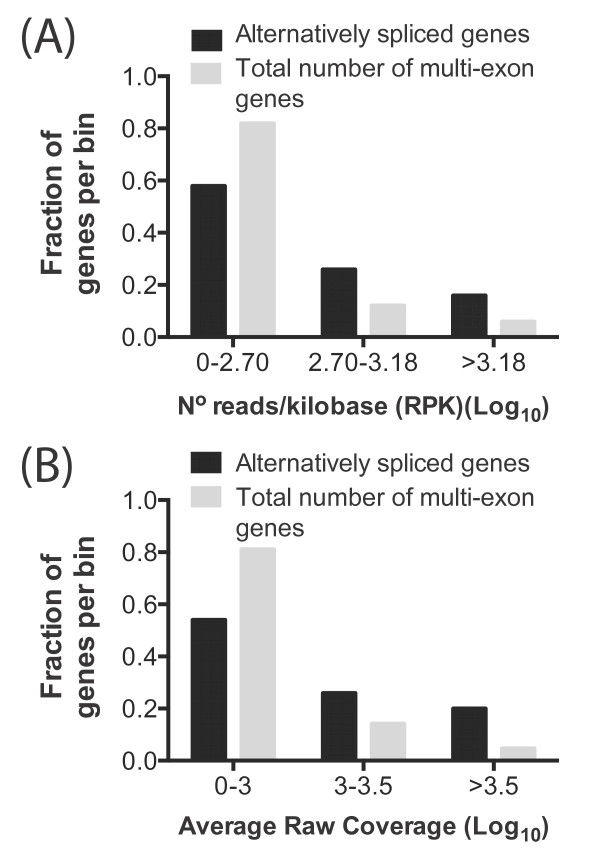
**The ability to detect alternatively spliced isoforms using RNA-seq data is dependent on read coverage.** Shown are fraction of alternatively spliced (from a total of 50) (Black bars) and multi-exonic genes (from a total of 5,873)(Grey bars) identified in bins (**A**) grouped by the Log_10_ of the expression level as reads per kilobase (RPK) or (**B**) grouped by the Log_10_ of raw coverage. Alternative isoforms are more likely to be detected amongst highly expressed genes compared to genes with low expression. See also additional file 9 for the relationship between read coverage and full assembly of transcripts.

The limitation of our method for alternative isoform discovery is that junction read coverage of some genes may be insufficient to detect all mRNA isoforms. Therefore to determine the effect of gene expression level on the sensitivity of our alternative isoform detection method, we assessed whether the genes with the highest read coverage exhibited a frequency of alternative splicing different from that of genes with lower coverage. Briefly, we first identified all single-exon protein-encoding PASA transcripts; 3,958 were confirmed as single exon genes and did not have any alternative isoforms and were not considered further. The remaining 5,873 set of non-redundant multi-exonic genes was binned based on read coverage. We then determined the fraction of alternatively spliced genes in each bin (Figure 5A-B). We found that the ability to detect alternatively spliced genes was dependent on expression level, with alternatively spliced genes significantly enriched in the top bin (Chi-square *p= 0.0001*). To differentiate major and minor isoforms, we compared their relative expression levels (Additional file 9) and designated the most abundant as the major isoform. We expect that with transcriptomes from different host infection models, different *Toxoplasma* growth stages (bradyzoites, tachyzoites) and strains, and with higher RNA-seq read coverage, more alternatively spliced *Toxoplasma* genes will be detected in the future.

### Differential isoform usage amongst different *Toxoplasma* strains

Differential isoform usage has previously been shown to be a determinant in disease progression in mice [45], humans [46], and parasite virulence [8]. Having detected alternatively spliced genes, we evaluated differential isoform usage amongst 3 clonal strains of *Toxoplasma* prevalent in Europe and North America (two type II strains Pru and ME49 and a type I strain RH) using the mixture-of-isoforms (MISO) algorithm [47]. MISO leverages the number of reads aligning to exons and exon junctions, taking into account the insert lengths for paired-end sequencing, to compute the percentage spliced in (PSI) value, which is the relative fraction of reads supporting the inclusion isoform [47]. The percentage spliced in (PSI) values for select genes are presented in Table 1. Based on the PSI values, there is evidence for alternative isoform usage by the *Toxoplasma* strains.

**Table 1:** **Percentage spliced in (PSI) (shown as a fraction) values for some of the alternatively spliced transcripts in the three clonal strains of*Toxoplasma gondii***

**Gene ID**	**Description**	**ME49**	**Pru**	**RH**
TGME49_053370	Roptry neck 4 L1 homologue	0.41	0.23	0.16
TGME49_008740	microneme protein, putative	0.28	0.31	0.46
TGME49_078510	protein phosphatase 2C, putative	0.71	0.72	0.61
TGME49_038230	serine/threonine protein phosphatase, putative	0.48	0.08	0.09
TGME49_112660	Hypothetical protein	0.50	0.55	0.05
TGME49_097470	Myosin light chain 2, putative	0.34	0.43	0.02

**Figure 6:**
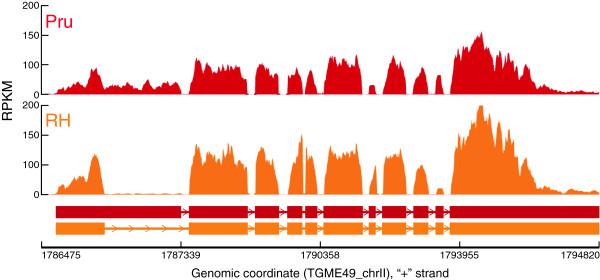
**Different isoforms of alternatively spliced transcripts are differentially expressed in diverse *Toxoplasma *strains.** Shown is RNA-seq read pile up (histogram) on the exons (horizontal bars) of alternative isoforms of TGME49_097470 gene that show differential isoform usage between a type II (Pru) and a type I (RH) strain. The predominant isoform for the TGME49_097470 gene in RH has identical gene model and protein sequence as that predicted in ToxoDB.

However, because PSI is based on the number of reads, which varies between samples, the PSI value will be more informative for each sample and not between samples. To correct for the variations between samples when comparing alternative splicing, MISO uses the Bayes factor to calculate the odds of differential splicing occurring. We illustrate this by comparing splicing in Pru and RH strains, two commonly used lab strains representing type II and type I strains, respectively, and present a summary of genes, with at least 5 times (Bayes factor of 5) probability of being spliced in either strain, together with the corresponding values of reads supporting each isoform (Table 2). As further evidence for the presence of the different transcript variants, we show a piling of RNA-seq reads on a pair of isoforms in Pru and RH (Figure 6a-b). The alternatively spliced transcripts are from genes encoding a protein phosphatase, myosin light chain, rhoptry neck, a micronemal, a RNA debranching enzyme, and RNA-binding proteins, amongst others. Because some of these genes are known to play some role in parasite invasion of cells, and alternative splicing leads to two protein isoforms, there is a potential for this variation to alter parasite virulence. For example, the one isoform of the micronemal protein is 769 amino acids long [48] while the alternative isoform has only 727 amino acids, which would possibly affect the biology of the parasite e.g. host cell invasion.

**Table 2:** Differential isoform usage between Pru and RH strains

**Gene**	**Pru**	**RH**
TGME49_008740	(0,0):3272,(0,1):62,(1,0):14,(1,1):275	(0,0):9355,(0,1):99,(1,0):73,(1,1):799
TGME49_112660	(0,0):580,(0,1):2,(1,0):4,(1,1):68	(0,0):667,(0,1):23,(1,1):74
TGME49_078510	(0,0):2647,(0,1):15,(1,0):15,(1,1):178	(0,0):4612,(0,1):18,(1,0):5,(1,1):225
TGME49_097470	(0,0):1648,(0,1):12,(1,0):22,(1,1):140	(0,0):3796,(0,1):19,(1,1):297

(0,0):**x** indicates x reads align to both isoforms but are not used in support of either isoform for a variety of reasons including reads aligning to exons far removed from the spliced site, (0,1)**:x**, x reads support the second isoform but not the first, (1,0):**x** indicate x reads support the first isoform and not the second (1,1):**x**, x reads supporting both isoforms. Missing values for RH indicate the absence of reads supporting the alternative isoform. The gene descriptions are shown in Table 1.

## Conclusions

In the current study, we have used approximately 270 million 40 bp paired-end RNA-seq reads to reconstitute *Toxoplasma* full-length transcripts *de novo*, evaluated the level of alternative splicing and accuracy of gene model prediction of ToxoDB genes. We have discovered a higher percentage of ToxoDB gene model inaccuracies, especially in UTR and terminal exon regions, than previously reported [7] and report on alternative transcript isoforms as opposed to the single transcript per gene currently reported in Toxodb. Our results indicate that RNA-seq is a robust and a relatively cheap method compared to full-length cDNA sequencing that can be used to annotate *Toxoplasma* genes and transcripts. However, as indicated by our results, this method requires high RNA-seq read coverage. Some of the alternatively spliced transcripts that we have identified are products of genes known to play some role in *Toxoplasma* biology e.g. attachment to and entry into the host cell. However, the relevance of each of these alternative transcript isoforms in parasite virulence needs further investigation. Based on our preliminary analysis, some of the transcripts identified are putative long intergenic non-coding RNAs but further conclusive analysis is necessary. Once the genome sequencing of the *Toxoplasma gondii* strains currently underway is completed, a strategy similar to that employed for the identification of mouse and zebrafish lincRNAs [24,43,49], may be employed to determine if some of these transcripts are lincRNAs.

## Methods

### Generation of *Toxoplasma* mRNA and RNA-sequencing

The *Toxoplasma* parasite strains (types II PruA7 and ME49, and type I RH) used in this experiment and its maintenance has previously been described [50-53]. Bone marrow derived macrophages (BMDM) obtained from 25 6–10 weeks old AxB/BxA recombinant inbred mice and their progenitors (AJ and C57BL/6J) were seeded in 12 well plates and infected with *Toxoplasma* at a multiplicity of infection (MOI) of 1.3 for 8 hours. Additionally, bone marrow derived macrophages from C57BL/6J mice were infected with either ME49 or RH *Toxoplasma* strains for 24 hours. Total RNA excluding mirRNA was extracted using the Qiagen RNeasy Plus kit (Qiagen, USA). Integrity, size distribution and concentration of RNA were then checked using the Agilent 2100 Bioanalyser.

The RNA samples were then processed for high-throughput sequencing according to standard Illumina protocols. Briefly, after mRNA pull-down from total RNA using Dynabeads mRNA Purification Kit (Invitrogen), mRNA was fragmented into 200–400 base pair-long fragments and reverse transcribed into cDNA before Illumina sequencing adapters were attached to each end. Samples were barcoded (each barcode was unique to each sample) and 4 samples were multiplexed in a single lane on an Illumina sequencing flow cell for paired end sequencing on an Illumina HiSeq 2000 machine. Our preliminary RNA-seq experiments of infected BMDM have shown that 4 samples per lane still results in enough read density for reliable gene expression analysis while significantly reducing the cost of sequencing. The Illumina sequencing pipeline performed primary data acquisition, determined base calls and calculated confidence scores.

### *De novo* transcript assembly and annotation

For the purposes of *de novo* transcript assembly, we concatenated only the reads obtained from the Pru-infected bone marrow derived macrophages (27 samples) before using these in Trinity. Because the RNA-seq reads obtained above originate from human (the *Toxoplasma* parasite used in this experiment was grown on human foreskin fibroblast), mouse and *Toxoplasma* genes, we initially aligned all the reads to mouse (*mm*9) and human (*hg*18) genomes using Bowtie (v0.12.7) and Tophat (v2.0.0) with the default settings. The resulting non-aligned reads were considered *Toxoplasma* reads and were used in Trinity [18] for transcript assembly. Trinity is a compilation of 3 distinct programs, each relying on the result of the preceding one to assemble full transcripts independent of a reference genome (for detailed description see Grabherr *et al*. [18]). Initially Trinity builds a k-*mer* (in our case 25 *mer*) library using the RNA-seq data, removes error-containing k-*mer* and singletons from the library, selects the most abundant k-mer as a seed and extends the seed on both directions by finding the next abundant k-*mer* with a k-1 *mer* overlap with the current contig terminus until the seed cannot be extended any further. Once a k-*mer* is used in a seed, it is removed from the k-*mer* library (i.e. a k-*mer* is used only once). We used Trinity (trinityrnaseq_r2012-06-08) default settings plus the jaccard clip option and the maximal heap space.

Next we used the Program to Assemble Spliced Alignment (PASA) [29] (PASA-r2011_05_20) with its default settings, to assemble contigs based on the ME49 genome (7.2 release). PASA aligned the Trinity assembled contigs to the ME49 genome using the Genome Mapping and Alignment Program (GMAP) [53] (gmap-2007-09-28), filtered invalid contigs (those most likely arising from sequencing and Trinity assembly errors), and reconstructed more complete transcripts from the Trinity contigs by reporting only the single best alignment. To achieve this, we constrained our analysis to Trinity contigs with at least 90% of their length sharing at least 95% similarity with the ME49 genomic sequence, and for which all the inferred exon-intron boundaries have canonical splice sites. The use of PASA to assemble full transcripts based on RNA-seq data has previously been described [29-31]. Next, we searched for intersections between the PASA transcripts and ME49 gene coordinates in BEDtools followed by alignment of overlapping PASA and ME49 protein sequences to analyze inaccuracies in gene models.

### Assessing alternative splicing and isoform usage

Once we annotated the *Toxoplasma* genes based on our PASA alignments, we assembled all the exon coordinates and chromosomal locations relative to the ME49 genome in gene transfer file format (gtf). We used this file in MISO [47] to quantify the expression level of, isoform usage of, and pile reads to the alternatively spliced genes amongst three *Toxoplasma gondii* clonal strains.

### Availability

Raw short read RNA-seq data have been submitted to the ToxoDB.

## Competing interests

The authors declare that they have no competing interests.

## Authors' contribution

JPJS and MH conceived of the study and wrote the manuscript. MH and KDJ prepared and processed the PA7 RNA and sequencing data. BH carried out the PASA genome annotation. MM contributed the RNA-seq data for ME49 and RH. All the authors have read and approved the manuscript.

## Supplementary Material

Additional file 1 Nucleotide sequences of all the PASA transcripts. Click here for file

Additional file 2 A bed file with the genome coordinates of all the PASA transcripts. Click here for file

Additional file 3 Sequences for Trinity contigs having invalid genome alignment in PASA but aligning to the ME49 genome in Blast. Click here for file

Additional file 4 The classes of transcripts obtained at different stages of our analysis pipeline. Click here for file

Additional file 5 **Blast search results of Trinity contigs rejected in PASA against ME49 known genes.**) Click here for file

Additional file 6 Evidence from ToxoDB showing splice junction tracks supporting the fusion of TGME49_005240 and TGME49_005230. Click here for file

Additional file 7 Identities of PASA transcripts and the ME49 genes they overlap in addition to the average read coverage. Click here for file

Additional file 8 **A figures showing the correlation between ability to reconstruct full transcripts of *Toxoplasma *genes and (A) expression (represented as reads per kilobase) and (B) RNA-seq read coverage.** We binned the transcripts based on their RPK or raw read coverage values and we show the fractions of fully assembled transcripts in each bin (from a total of 2073 fully assembled genes). For this figure, fully assembled transcripts were defined as those producing ORFs that matched the ToxoDB proteins both in length and sequence (2073 total). Click here for file

Additional file 9 Table showing novel ME49 genes. Click here for file

Additional file 10 Identities of PASA transcripts supporting alternative splicing classes and their relative expression values in 27 sequenced samples. Click here for file
