# Supplementary figures and images for: De novo reconstruction of the Toxoplasma gondii transcriptome improves on the current genome annotation and reveals alternatively spliced transcripts and putative long non-coding RNAs
Source: BMC Genomics. 2012 Dec 12;13:696. doi: 10.1186/1471-2164-13-696 (PMC3543268; doi:10.1186/1471-2164-13-696)

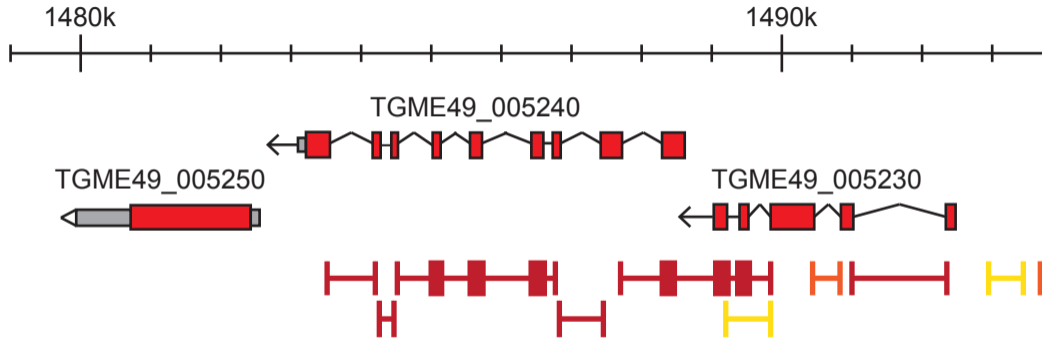

Supplement: Additional file 6 — Evidence from ToxoDB showing splice junction tracks supporting the fusion of TGME49_005240 and TGME49_005230. [file 1471-2164-13-696-S6.pdf]

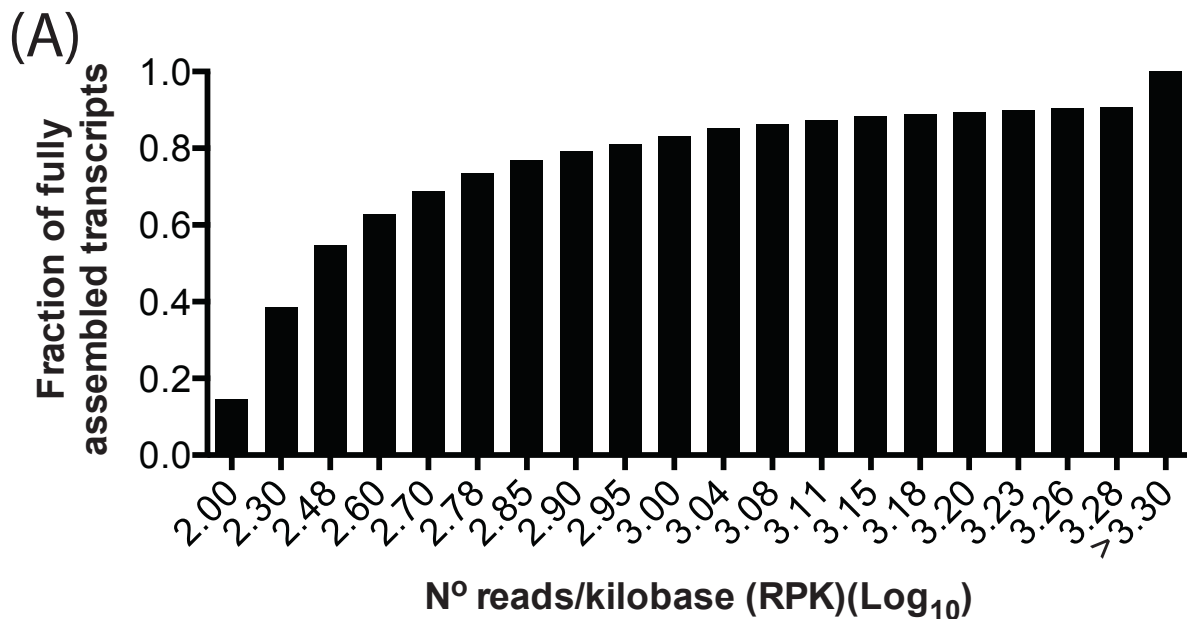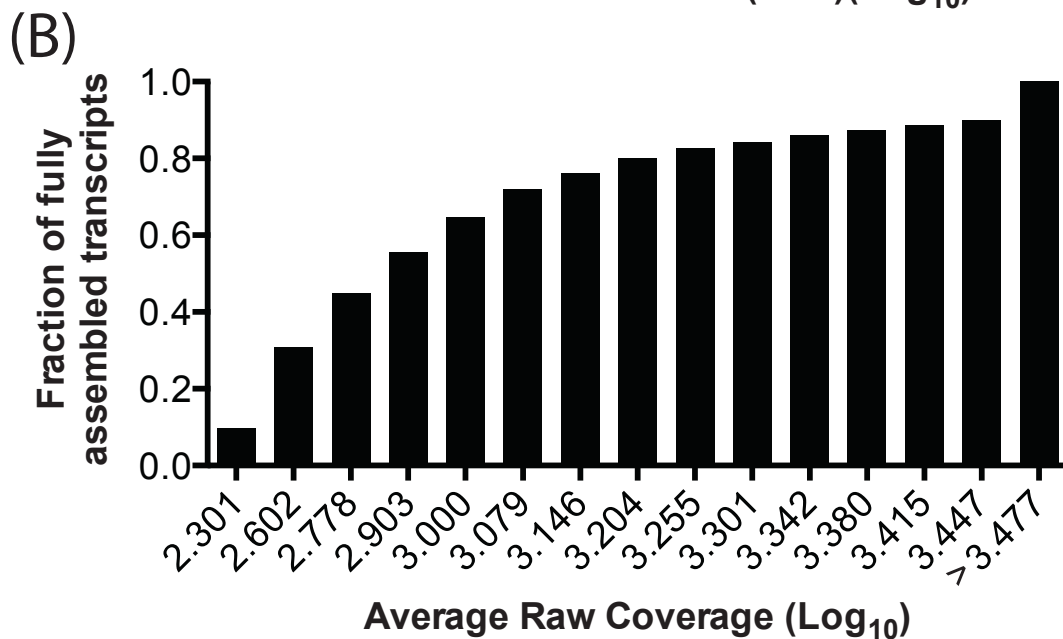

Supplement: Additional file 8 — A figures showing the correlation between ability to reconstruct full transcripts of Toxoplasma genes and (A) expression (represented as reads per kilobase) and (B) RNA-seq read coverage. We binned the transcripts based on their RPK or raw read coverage values and we show the fractions of fully assembled transcripts in each bin (from a total of 2073 fully assembled genes). For this figure, fully assembled transcripts were defined as those producing ORFs that matched the ToxoDB proteins both in length and sequence (2073 total). [file 1471-2164-13-696-S8.pdf]
