# Supplementary material for: De novo reconstruction of the Toxoplasma gondii transcriptome improves on the current genome annotation and reveals alternatively spliced transcripts and putative long non-coding RNAs
Source: BMC Genomics. 2012 Dec 12;13:696. doi: 10.1186/1471-2164-13-696 (PMC3543268; doi:10.1186/1471-2164-13-696)
Supplement: Additional file 9 — Table showing novel ME49 genes. [file 1471-2164-13-696-S9.docx]

| PASA ID | Protein matched in Blastx | Protein description |
| --- | --- | --- |
| S11097;_asmbl_11157;_S11097_asmbl_11157 | TGGT1_082090 | Hypothetical protein |
| S11720;_asmbl_11781;_S11720_asmbl_11781 | TGVEG_064280 | Hypothetical protein |
| S13765;_asmbl_13835;_S13765_asmbl_13835 | TGVEG_098940 | Hypothetical protein |
| S14238;_asmbl_14309;_S14238_asmbl_14309 | TGVEG_101760 | Hypothetical protein |
| S14394;_asmbl_14466;_S14394_asmbl_14466 | TGGT1_098810  TGVEG_037860 | Hypothetical protein  Hypothetical protein |
| S14421;_asmbl_14493;_S14421_asmbl_14493 | TGGT1_098990  TGVEG_038030 | Hypothetical protein  Hypothetical protein |
| S15499;_asmbl_15573;_S15499_asmbl_15573 | TGVEG_003790 | Hypothetical protein |
| S4242;_asmbl_4267;_S4242_asmbl_4267 | TGVEG_029770 | Hypothetical protein |
| S4490;_asmbl_4515;_S4490_asmbl_4515 | TGVEG_107950 | Hypothetical protein |
| S5476;_asmbl_5505;_S5476_asmbl_5505 | TGGT1_051870  TGVEG_092430 | Hypothetical protein  Hypothetical protein |
| S5743;_asmbl_5775;_S5743_asmbl_5775 | TGVEG_090490 | Hypothetical protein |
| S5784;_asmbl_5816;_S5784_asmbl_5816 | TGGT1_049480  TGVEG_090070 | Hypothetical protein  Hypothetical protein |
| S6149;_asmbl_6187;_S6149_asmbl_6187 | TGVEG_103880 | Hypothetical protein |
| S746;_asmbl_750;_S746_asmbl_750 | TGGT1_021300  TGVEG_040090 | Hypothetical protein  Hypothetical protein |
| S779;_asmbl_783;_S779_asmbl_783 | TGVEG_039890 | Hypothetical protein |
| S8868;_asmbl_8920;_S8868_asmbl_8920 | TGVEG_079120 | Hypothetical protein |
| S8872;_asmbl_8924;_S8872_asmbl_8924 | TGVEG_079190 | Hypothetical protein |
| S9617;_asmbl_9671;_S9617_asmbl_9671 | TGVEG_021860 | Hypothetical protein |

**Additional file 8**: Shown are the protein products of PASA transcripts that did not overlap with any known ME49 gene and the *Toxoplasma* proteins they aligned to after blastx search.
