# Supplementary material for: De novo reconstruction of the Toxoplasma gondii transcriptome improves on the current genome annotation and reveals alternatively spliced transcripts and putative long non-coding RNAs
Source: BMC Genomics. 2012 Dec 12;13:696. doi: 10.1186/1471-2164-13-696 (PMC3543268; doi:10.1186/1471-2164-13-696)
Supplement: Additional file 10 — Identities of PASA transcripts supporting alternative splicing classes and their relative expression values in 27 sequenced samples. [file 1471-2164-13-696-S10.docx]

| **Description** | **No.** |
| --- | --- |
| Total number of paired-end RNA-seq reads used | ~270 million |
| Total Trinity fragments | 29,294 |
| Number of fragments with valid alignments in PASA | 23,898 |
| Number of PASA assembled transcripts | 16,462 |
| PASA transcripts with ToxoDB gene overlap | 13,533 |
| PASA transcripts with ORFs matching ToxoDB proteins in Blastx | 9,592 |
| PASA transcripts with no ME49 gene overlap but with Blastx match | 239 |
| Potential ncRNA | 18 |
| Alternatively spliced transcripts | 50 |
